# Diversity of endophytic mycobiota of tropical tree *Tectona grandis* Linn.f.: Spatiotemporal and tissue type effects

**DOI:** 10.1038/s41598-017-03933-0

**Published:** 2017-06-16

**Authors:** Dheeraj K Singh, Vijay K Sharma, Jitendra Kumar, Ashish Mishra, Satish K Verma, Thomas N Sieber, Ravindra N Kharwar

**Affiliations:** 1 Mycopathology and Microbial Technology Laboratory, Department of Botany, Banaras Hindu University (BHU), Varanasi, 221005 India; 2 ETH Zurich, Institute of Integrative Biology, Forest Pathology and Dendrology, 8092 Zurich, Switzerland

## Abstract

Fungal endophytes were isolated from leaf, bark and stem of *Tectona grandis* Linn.f. sampled at four geographical locations in winter, summer and monsoon seasons. The recovered 5089 isolates were assigned to 45 distinct morphotypes based on morphology. The sequences of the internal transcribed spacers (ITS) of the nrDNA of some morphotypes were identical, but morphological differences were strong enough to consider these morphotypes as separate species. Forty-three morphotypes were assigned to ascomycotina and two to basidiomycotina. Ascomycotina was the predominating group with 99.7% of total isolates followed by basidiomycotina with only 0.3% of total isolates. *Diaporthe* (*Phomopsis*) species dominated the communities independently on tissue type, location or season. More than 60% of the examined tissue pieces were colonized by members of this species complex. While these endophytes are ubiquitous others were tissue or location specific. Tissue type had the strongest effect on the species evenness of the endophytic assemblage followed by geographical location and season. However, Shannon-Wiener index (*H’*) significantly (p ≤ 0.001) varied with all three factors i.e. season, location and tissue type. Leaves supported the highest diversity across all the seasons and locations. In conclusion, all the three factors together determined the structure of endophytic mycobiota assemblage of *T*. *grandis*.

## Introduction

The start of endophytes research dates back to year 1866 when a German botanist Anton de Bary, first introduced the term “Endophytes” for any *in planta* microorganism^1^. In strict sense, fungal endophytes are fungi that spend either full or a considerable part of their life inside living plant tissues without causing any visible harm^2^. After decades of research on fungal endophytes, it is now clear that they are unexceptionally present in all taxonomic groups of the plant kingdom, vegetation types (alpine to tropical) and ecological types (hydrophytes to xerophytes) in great diversity^3–5^. Discovery of high endophyte diversity in trees^3, 6^ has led to a surge in research efforts in this direction especially on trees growing in tropical regions. Some tropical tree species have been reported to host a hyper diverse endophyte community^3, 7^. Exploration of tropical woody tree endophyte diversity will definitely improve global fungal diversity estimates. Due to a plethora of factors concomitantly affecting endophyte assemblage in a tree, it is hard to assign specific role to one particular factor in this context^3, 8^. But the studies covering different regulating factors with appropriate sampling size will definitely improve our understanding about their roles. Isolation of endophytes is also important in view of their potential as prolific source of novel compounds with applications in pharmaceutical, agricultural and food industries^9–11^. Due to their unique and diverse niche different bioactive secondary metabolites can be obtained from the same endophyte species as their metabolite spectrum changes with change in environment, geography, host and tissue type^12^. Consequently, the study of endophyte communities in different tissues and plants is essential to harness the maximum benefits of this great resource.

Due to diverse ecological communities in wide-ranging environmental regimes, India is a suitable place for the assessment of endophytes and factors responsible for shaping their assemblage. *Tectona grandis* Linn.f. (Teak), family Verbenaceae, is a major tree of tropical regions and native to India. Teak can grow under a wide range of climatic and edaphic conditions. All parts of teak, including, bark, leaf, stem, root, flower and seed, are traditionally used in folklore medicine to cure various diseases. Its good pharmacological potentials, such as, cytotoxic (ellagic acid), antibacterial (juglone), antifungal (deoxylapachol and tectoquinine), antioxidant (gallic acid, ferulic acid and quercetin), anti-diabetes, anti-inflammatory and tocolytic effects have been well documented^13, 14^. The above mentioned outstanding properties of *T*. *grandis* and the possibility of horizontal gene transfer from host to endophytes motivated us to select this plant as a study material. Teak is one of the most studied tropical trees with regard to its ecology and application in forestry, however, it has rarely been subjected to endophytic study^15, 16^. Hitherto, there is not a single report available on *Tectona* which includes such disparate tissue types and sampling sites with different climatic conditions and seasonal variability.

The aim of the present work was to study geographical and seasonal variations in fungal endophyte assemblage of *T*. *grandis* in three different tissues. Fungal endophytes were identified using both morphological and molecular tools.

## Results

### Diversity of endophytic mycobiota in *Tectona grandis*

The 5089 fungal isolates obtained from the 8100 tissue segments could be assigned to 45 OTUs (operational taxonomic units referring to different morphological types) based on culture morphology. All, except for two OTUs, sporulated in culture and could be identified to the genus or species level. Molecular identification based on ITS sequences corroborated morphological identification in almost all cases (Table 1). ITS sequencing revealed names for non-sporulating OTUs too. Interestingly, many OTUs possessed almost identical ITS sequences (i.e. *Tectona grandis* endophytes TGE1 and TGE16, TGE2 and TGE3, TGE8 and TGE27, TGE9 and TGE10, TGE11 and TGE20, TGE18 and TGE19, TGE28 and TGE30), although the OTUs were distinctly different in micromorphology, culture morphology and matched with different reference strains of NCBI database. Due to these differences TGEs with almost identical ITS sequences were considered separate species. To minimize confusion between morphological and molecular identity bellow species level, ITS based name is given in parenthesis wherever required.

**Table 1:** Morphological and molecular (based on nrITS1 sequencing) identification of different OTUs, their closest match from NCBI database with their accession number, similarity (%ID) and query coverage (%QC).

OTU acronym	GenBank accession No.^2^	Morphological identification^1,2^	Closest NCBI match^1^	ID/QC (%)	Reference accession No.
TGE1	KF688116	***Paecilomyces*** **sp**.**1**	*Paecilomyces variotii*	100/99	KY019238
TGE2	KF688118	***Alternaria*** **sp**.**1**	*Alternaria* sp.	100/100	HQ914881
TGE3	KF910776	***Alternaria*** **sp**.**2**	*Alternaria* sp.	99/99	KP027305
TGE4	NS	***Aspergillus flavus***			
TGE5	NS	***Aspergillus fumigatus***			
TGE6	NS	***Aspergillus niger***			
TGE7	KU519494	***Aspergillus*** **sp**.**1**	*Aspergillus viridinutans*	98/100	NR135409
TGE8	KF688119	***Aspergillus*** **sp**.**2**	*Aspergillus versicolor*	100/100	KX776009
TGE9	JX951183	***Chaetomium globosum***	*Chaetomium globosum*	100/99	JF826006
TGE10	KF688114	***Chaetomium*** **sp**.	*Chaetomium* sp.	100/100	HM535379
TGE11	KF688120	***Colletotrichum gloeosporioides***	*Colletotrichum gloeosporioides*	99/100	KX347456
TGE12	KF688125	***Corynespora cassiicola***	*Corynespora cassiicola*	100/100	KY290563
TGE13	KU519495	***Emericella*** **sp**.	*Aspergillus quadrilineatus*	100/100	KX683008
TGE14	KF688115	***Gibberella baccata***	*Fusarium* sp.	100/99	KM066585
TGE15	KU519496	***Fusarium*** **sp**.**1**	*Albonectria rigidiuscula*	100/100	KX788159
TGE16	KF688112	***Paecilomyces*** **sp**.**2**	*Paecilomyces variotii*	99/100	JX231004
TGE17	KF688126	***Ceratobasidium*** **sp**.	*Ceratobasidium* sp.	99/100	HM623627
TGE18	KF910777	**MS1**	*Pseudofusicoccum adansoniae*	99/100	KF766220
TGE19	KF910778	**MS2**	*Lasiodiplodia theobromae*	99/100	KM508495
TGE20	KF688122	***Colletotrichum*** **sp**.	*Colletotrichum siamense*	100/100	KY242353
TGE21	NS	***Penicillium*** **sp**.			
TGE22	KF688130	***Phomopsis*** **sp**.**1**	*Diaporthe* sp.	100/100	KU663500
TGE23	KF910772	***Phomopsis*** **sp**.**2**	*Diaporthe amygdali*	99/100	KF308686
TGE24	KF910766	***Phomopsis*** **sp**.**3**	*Phomopsis longicolla*	99/100	KP174768
TGE25	KF910765	***Lasiodiplodia theobromae***	*Lasiodiplodia theobromae*	100/100	KX506786
TGE26	KU512642	***Fusarium*** **sp**.**2**	*Fusarium incarnatum*	100/100	KX184815
TGE27	KF910774	***Aspergillus*** **sp**.**3**	*Aspergillus versicolor*	99/100	LT594460
TGE28	KF910773	***Cercospora*** **sp**.**1**	*Cercospora gerberae*	99/100	KX347479
TGE29	KF688117	***Phomopsis*** **sp**.**4**	*Diaporthe* sp.	99/100	KC145847
TGE30	KF688113	***Cercospora*** **sp**.**2**	*Cercospora nicotianae*	99/100	KM485926
TGE31	KF688123	***Periconia*** **sp**.**1**	*Periconia* sp.	100/100	JF817312
TGE32	KF910767	***Botryosphaeria*** **sp**.	*Lasiodiplodia pseudotheobromae*	100/100	KY583266
TGE33	KF910769	***Diaporthe*** **sp**.	*Diaporthe* sp.	99/100	KU375727
TGE34	KF688129	***Guignardia*** **sp**.	*Phyllosticta elongata*	100/100	KX424992
TGE35	KF910770	***Glomerella*** **sp**.	*Colletotrichum acutatum*	99/100	KX347475
TGE36	KF688121	***Phomopsis*** **sp**.**5**	*Phomopsis* sp.	99/100	DQ235667
TGE37	KU512643	***Lasiodiplodia*** **sp**.	*Diplodia seriata*	100/100	KY620311
TGE38	KF910775	***Fusarium*** **sp**.**3**	*Albonectria rigidiuscula*	100/99	KY024396
TGE39	KF688127	***Hypoxylon*** **sp**.	*Hypoxylon fragiforme*	99/100	KU317724
TGE40	KF688128	***Coprinellus*** **sp**.	*Coprinellus* sp.	99/99	GQ249274
TGE41	JX951186	***Periconia*** **sp**.**2**	*Periconia* sp.	98/100	JF817328
TGE42	KF910768	***Pyrenochaeta*** **sp**.	*Pyrenochaeta* sp.	99/99	GQ895137
TGE43	KF688124	***Phomopsis*** **sp**.**6**	*Phomopsis* sp.	100/100	KX401432
TGE44	KF910771	***Curvularia*** **sp**.	*Curvularia* sp.	100/100	HE861827
TGE45	KF910779	***Alternaria*** **sp**.**3**	*Alternaria* sp.	100/100	KM051397

^1^Species names according to the Index fungorum (http://www.indexfungorum.org/Names/Names.asp; accessed March 25, 2017). ^2^MS1 and 2 = Mycelia-Sterilia; NS = not sequenced, TGE = *Tectona grandis* endophyte.

**Figure 1:**
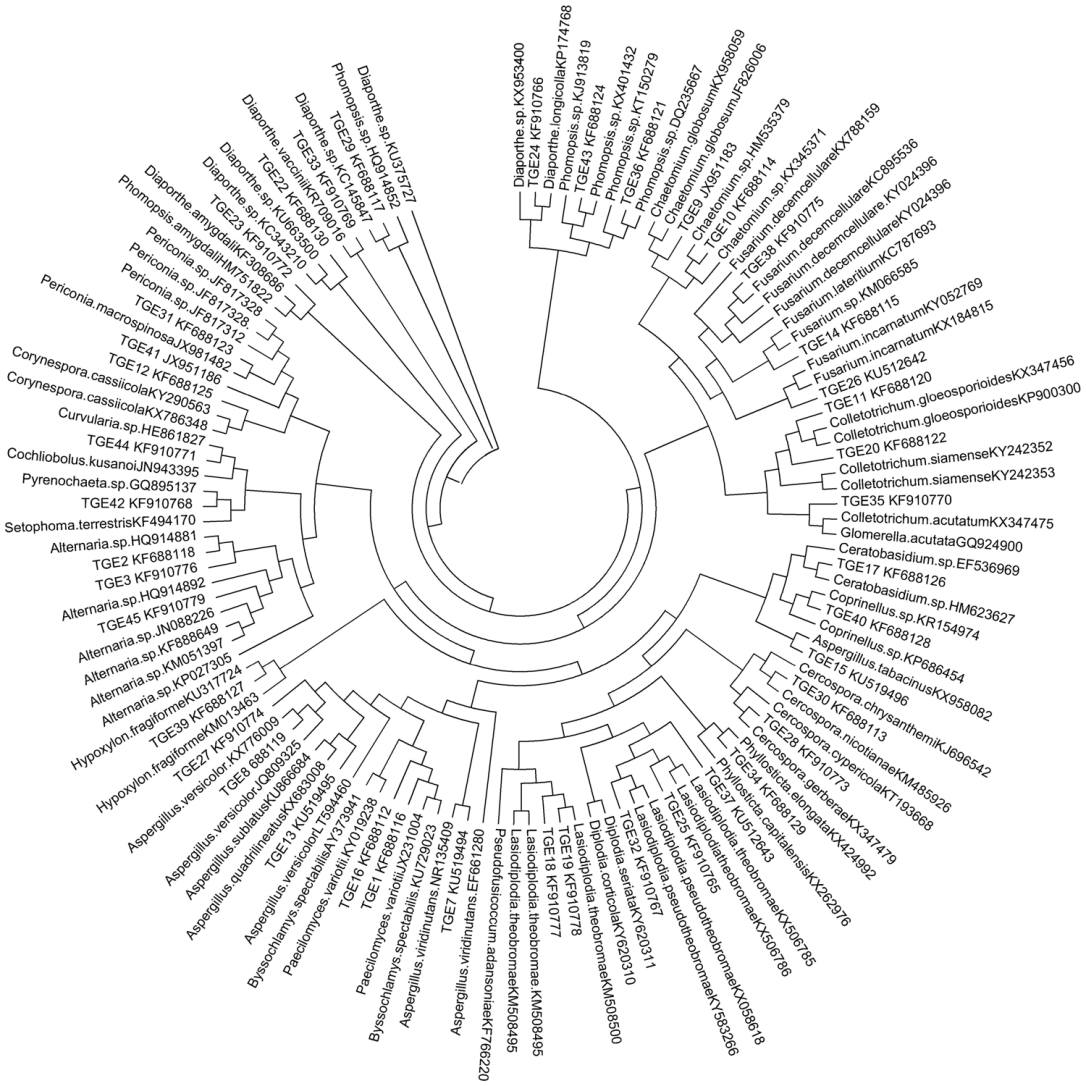
Phylogenetic tree constructed by neighbour-joining method using nrDNA ITS sequences generated during this study (as TGE) and sequences retrieved from GenBank using p-distance for nucleotides and the pairwise gap deletion choice.

The dendrogram created using ITS sequences of endophytic OTUs and reference taxa retrieved from NCBI database shows that the endophyte assemblages of *T*. *grandis* include representative taxa of the ascomycotina and basidiomycotina (Fig. 1). All The members of ascomycotina and basidiomycotina formed eighteen different clades in the dendrogram.

Overall frequency of colonization of fungal endophytes in *T*. *grandis* tissues was 62.83%. *Diaporthe* (*Phomopsis*) spp. dominated the endophytic assemblage in teak tissues. *Phomopsis* sp.3 (*Phomopsis longicolla*) was the most frequent species (21.12%) followed by *Phomopsis* sp.1 (*Diaporthe* sp.) (12.38%). *Colletotrichum gloeosporioides* was the third most abundant species with 11.08% of dominance. Eighteen other OTUs showed abundance ranging from 1% to 7.8% and collectively amounted to almost half of the isolates (48.12%). The above mentioned 21 morphotypes with overall share of 92.7% were the predominating taxa in the *T*. *grandis* endophyte assemblage. Seven morphotypes with relative abundance from ≥0.5% to <1% were considered frequent taxa while the remaining 17 morphotypes whose relative abundance was <0.5% were considered as rare. Interestingly, *Chaetomium globosum* and *Guignardia* sp. (*Phyllosticta elongata*) were among frequent OTUs but were never isolated from bark and stem. Similarly, *Lasiodiplodia theobromae* was limited to location 2 (Loc2, Hathinala, Uttar Pradesh) only. Detail of colonization pattern of fungal endophytes in different seasons and tissue types of *T*. *grandis* at four geographical locations is given in Table 2

**Table 2:** Number, percent colonization frequency (%CF) and percent dominance (%D) of different fungal endophytes of *T*. *grandis*.

**Endophyte**	**Winter**												**Summer**												**Monsoon**												**Total**	**% CF**	**% D**
	**Loc1**			**Loc2**			**Loc3**			**Loc4**			**Loc1**			**Loc2**			**Loc3**			**Loc4**			**Loc1**			**Loc2**			**Loc3**			**Loc4**					
	**B**	**L**	**S**	**B**	**L**	**S**	**B**	**L**	**S**	**B**	**L**	**S**	**B**	**L**	**S**	**B**	**L**	**S**	**B**	**L**	**S**	**B**	**L**	**S**	**B**	**L**	**S**	**B**	**L**	**S**	**B**	**L**	**S**	**B**	**L**	**S**			
*Phomopsis* sp.3	21	36	30	24	30	27	26	36	34	36	40	37	17	26	23	21	27	27	23	29	27	30	34	30	25	34	29	29	32	32	29	35	34	33	37	35	**1075**	**13**.**2716**	**21**.**1240**
*Phomopsis* sp.1	0	26	23	0	29	27	0	29	26	0	33	29	0	23	17	0	24	16	0	26	20	0	24	30	0	28	24	0	30	27	0	32	28	0	30	29	**630**	**7**.**7778**	**12**.**3796**
*Colletotrichum gloeosporioides*	0	26	20	0	28	23	0	29	24	0	30	23	0	20	17	0	22	14	0	28	19	0	33	21	0	24	22	0	26	24	0	25	24	0	22	20	**564**	**6**.**9630**	**11**.**0827**
MS1	10	13	11	7	12	8	11	12	14	13	14	19	7	10	8	5	12	6	7	10	13	10	13	14	13	15	15	7	14	10	14	12	14	5	6	14	**398**	**4**.**9136**	**7**.**8208**
*Gibberell baccata*	22	5	0	29	0	0	35	0	0	30	4	0	20	4	0	22	2	0	26	3	0	26	4	0	24	8	0	26	3	0	30	10	0	36	7	0	**376**	**4**.**6420**	**7**.**3885**
MS2	5	13	9	6	9	8	8	9	9	9	11	10	5	7	6	6	8	8	8	9	8	8	10	10	11	14	9	6	12	8	10	14	9	9	13	11	**325**	**4**.**0123**	**6**.**3863**
*Alternaria* sp.1	4	6	7	0	7	9	0	6	9	5	7	8	3	4	5	0	4	6	0	5	7	4	5	6	6	8	8	0	8	9	5	8	10	7	8	10	**204**	**2**.**5185**	**4**.**0086**
*Colletotrichum* sp.	4	9	3	0	11	7	8	9	9	8	10	10	0	0	0	0	0	0	0	0	0	0	0	0	4	8	5	5	10	8	8	12	12	9	12	9	**190**	**2**.**3457**	**3**.**7335**
*Corynespora cassiicola*	9	0	6	21	24	9	0	0	0	0	0	0	0	0	0	0	0	0	0	0	0	0	0	0	13	0	7	26	31	16	0	0	0	0	0	0	**162**	**2**.**0000**	**3**.**1833**
*Fusarium* sp.1	6	0	4	6	0	6	8	0	7	9	0	7	3	0	2	2	0	1	5	0	4	8	0	6	8	0	5	6	0	6	10	0	9	12	0	7	**147**	**1**.**8148**	**2**.**8886**
*Cercospora* sp.2	0	0	0	0	0	0	6	6	6	9	7	10	0	0	0	0	0	0	3	3	4	7	6	9	0	0	0	0	0	0	6	8	7	8	9	7	**121**	**1**.**4938**	**2**.**3777**
*Phomopsis*sp.2	0	4	0	0	7	0	3	9	0	6	11	0	0	4	0	0	3	0	0	5	0	0	7	0	0	7	0	0	5	0	0	9	0	10	10	0	**100**	**1**.**2346**	**1**.**9650**
*Fusarium* sp.2	0	0	0	0	0	0	6	7	0	7	9	0	0	0	0	0	0	0	0	0	0	5	8	0	0	0	0	0	0	0	12	9	0	10	10	0	**83**	**1**.**0247**	**1**.**6310**
*Chaetomium globosum*	0	4	0	0	3	0	0	7	0	0	5	0	0	2	0	0	2	0	0	4	0	0	5	0	0	7	0	0	6	0	0	7	0	0	8	0	**60**	**0**.**7407**	**1**.**1790**
*Aspergillus flavus*	2	0	0	2	0	0	0	0	0	0	0	0	4	3	3	2	4	2	3	2	2	2	2	2	2	0	0	2	2	3	1	1	3	0	1	2	**52**	**0**.**6420**	**1**.**0218**
*Penicillium* sp.	1	0	1	0	4	0	2	1	3	2	1	1	0	1	0	1	2	0	2	1	1	2	1	1	1	2	1	0	2	0	1	2	2	1	1	1	**42**	**0**.**5185**	**0**.**8253**
*Aspergillus fumigatus*	1	0	1	0	0	1	1	0	2	2	0	1	1	0	1	2	0	1	1	0	1	3	0	2	3	1	1	2	1	0	2	1	2	3	2	1	**40**	**0**.**4938**	**0**.**7860**
*Guignardia* sp.	0	0	0	0	2	0	0	3	0	0	4	0	0	0	0	0	4	0	0	5	0	0	7	0	0	0	0	0	4	0	0	5	0	0	4	0	**38**	**0**.**4691**	**0**.**7467**
*Alternaria* sp.2	0	0	0	0	0	0	0	3	0	3	4	0	0	0	0	0	0	0	0	3	0	2	3	0	0	0	0	0	0	0	3	6	0	5	5	0	**37**	**0**.**4568**	**0**.**7271**
*Aspergillus niger*	2	0	0	2	0	0	1	0	2	1	0	1	5	1	0	2	0	3	0	0	2	2	0	3	1	0	0	1	0	0	1	0	3	3	0	1	**37**	**0**.**4568**	**0**.**7271**
*Botryosphaeria* sp.	0	0	0	7	0	6	0	0	0	0	0	0	0	0	0	4	0	6	0	0	0	0	0	0	0	0	0	8	0	6	0	0	0	0	0	0	**37**	**0**.**4568**	**0**.**7271**
*Phomopsis* sp.4	0	0	0	0	0	0	0	0	0	0	0	9	0	0	0	0	0	0	0	0	0	0	0	6	0	0	0	0	0	0	0	0	0	0	0	11	**26**	**0**.**3210**	**0**.**5109**
*Hypoxylon* sp.	0	0	0	0	0	0	4	0	0	0	0	0	0	0	0	0	0	0	4	0	0	0	0	0	0	0	0	0	0	0	7	0	0	9	0	0	**24**	**0**.**2963**	**0**.**4716**
*Periconia* sp.1	0	0	0	9	0	0	0	0	0	0	0	0	0	0	0	3	0	0	0	0	0	0	0	0	0	0	0	10	0	0	0	0	0	0	0	0	**22**	**0**.**2716**	**0**.**4323**
*Pyrenochaeta* sp.	0	0	0	0	0	0	0	0	7	0	0	0	0	0	0	0	0	0	6	0	0	0	0	0	0	0	0	0	0	0	0	0	9	0	0	0	**22**	**0**.**2716**	**0**.**4323**
*Curvularia* sp.	0	0	0	0	0	0	0	0	0	6	0	0	0	0	0	0	0	0	0	0	0	0	0	0	0	0	0	0	0	0	0	0	0	4	7	5	**22**	**0**.**2716**	**0**.**4323**
*Glomerella* sp.	7	0	0	0	0	0	0	0	0	0	0	0	5	0	0	0	0	0	0	0	0	0	0	0	9	0	0	0	0	0	0	0	0	0	0	0	**21**	**0**.**2593**	**0**.**4127**
*Phomopsis* sp.6	0	0	0	0	0	0	0	0	0	0	7	0	0	0	0	0	0	0	0	0	0	0	4	0	0	0	0	0	0	0	0	0	0	0	10	0	**21**	**0**.**2593**	**0**.**4127**
*Alternaria* sp.3	0	0	0	0	0	0	0	0	0	3	0	0	0	0	0	0	0	0	0	0	0	0	0	0	0	0	0	0	0	0	0	0	0	7	6	5	**21**	**0**.**2593**	**0**.**4127**
*Periconia* sp.2	0	0	0	0	0	0	0	6	0	0	0	0	0	0	0	0	0	0	0	4	0	0	0	0	0	0	0	0	0	0	0	9	0	0	0	0	**19**	**0**.**2346**	**0**.**3734**
*Aspergillus* sp.3	0	0	0	0	0	0	0	0	0	0	0	7	0	0	0	0	0	0	0	0	0	0	0	0	0	0	0	0	0	0	0	0	0	0	0	10	**17**	**0**.**2099**	**0**.**3341**
*Paecilomyces* sp.1	0	2	0	0	2	0	0	0	0	0	0	0	0	0	0	0	0	0	0	0	0	0	0	0	0	4	0	0	2	0	0	3	0	0	3	0	**16**	**0**.**1975**	**0**.**3144**
*Cercospora* sp.1	0	0	0	0	0	0	0	0	0	0	0	7	0	0	0	0	0	0	0	0	0	0	0	0	0	0	0	0	0	0	0	0	0	0	0	9	**16**	**0**.**1975**	**0**.**3144**
*Fusarium* sp.3	0	0	0	0	0	0	6	0	0	0	0	0	0	0	0	0	0	0	5	0	0	0	0	0	0	0	0	0	0	0	5	0	0	0	0	0	**16**	**0**.**1975**	**0**.**3144**
*Aspergillus* sp.2	0	0	0	0	0	0	0	0	3	0	0	2	0	0	0	0	0	0	0	0	0	0	0	3	0	0	0	0	0	0	0	0	4	0	0	3	**15**	**0**.**1852**	**0**.**2948**
*Chaetomium* sp.	0	0	0	0	0	0	0	3	0	0	4	0	0	0	0	0	0	0	0	0	0	0	0	0	0	0	0	0	0	0	0	4	0	0	4	0	**15**	**0**.**1852**	**0**.**2948**
*Emericella* sp.	0	2	0	0	3	0	0	0	0	0	0	0	0	0	0	0	0	0	0	0	0	0	0	0	0	5	0	0	5	0	0	0	0	0	0	0	**15**	**0**.**1852**	**0**.**2948**
*Phomopsis* sp.5	0	3	0	0	0	0	0	0	0	0	0	0	0	5	0	0	0	0	0	0	0	0	0	0	0	5	0	0	0	0	0	0	0	0	0	0	**13**	**0**.**1605**	**0**.**2555**
*Lasiodiplodia* sp.	0	0	4	0	0	0	0	0	0	0	0	0	0	0	3	0	0	0	0	0	0	0	0	0	0	0	6	0	0	0	0	0	0	0	0	0	**13**	**0**.**1605**	**0**.**2555**
*Diaporthe* sp.	0	0	0	3	0	0	0	0	0	0	0	0	0	0	0	3	0	0	0	0	0	0	0	0	0	0	0	5	0	0	0	0	0	0	0	0	**11**	**0**.**1358**	**0**.**2162**
*Ceratobasidium* sp.	0	0	0	0	0	0	7	0	0	0	0	0	0	0	0	0	0	0	0	0	0	0	0	0	0	0	0	0	0	0	3	0	0	0	0	0	**10**	**0**.**1235**	**0**.**1965**
*Lasiodiplodia theobromae*	0	0	0	0	0	0	0	0	0	0	0	0	0	0	0	0	0	0	0	0	0	0	0	0	0	0	0	0	6	0	0	0	0	0	0	0	**6**	**0**.**0741**	**0**.**1179**
*Coprinellus* sp.	0	0	0	0	0	0	3	0	0	0	0	0	0	0	0	0	0	0	0	0	0	0	0	0	0	0	0	0	0	0	3	0	0	0	0	0	**6**	**0**.**0741**	**0**.**1179**
*Paecilomyces* sp.2	0	3	0	0	0	0	0	0	0	0	0	0	0	0	0	0	0	0	0	0	0	0	0	0	0	0	0	0	0	0	0	0	0	0	0	0	**3**	**0**.**0370**	**0**.**0590**
*Aspergillus* sp.1	0	1	0	0	0	0	0	0	0	0	0	0	0	0	0	0	0	0	0	0	0	0	0	0	0	0	0	0	0	0	0	0	0	0	0	0	**1**	**0**.**0123**	**0**.**0197**
Total	**94**	**153**	**119**	**116**	**171**	**131**	**135**	**175**	**155**	**149**	**201**	**181**	**70**	**110**	**85**	**73**	**114**	**90**	**93**	**137**	**108**	**109**	**166**	**143**	**120**	**170**	**132**	**133**	**199**	**149**	**150**	**212**	**170**	**171**	**215**	**190**	**5089**	**62**.**8272**	**100**.**0**

Loc 1, 2, 3, 4 denotes location 1 to 4; B, L and S denotes bark, leaf and stem respectively.

. Among 45 fungal morphotypes, 43 (95.6%) belonged to ascomycotina and 2 (4.4%) to basidiomycotina. Of the total isolates, numbering 5089, 99.7% belonged to ascomycotina and the rest 0.3% to basidiomycotina.

**Figure 2:**
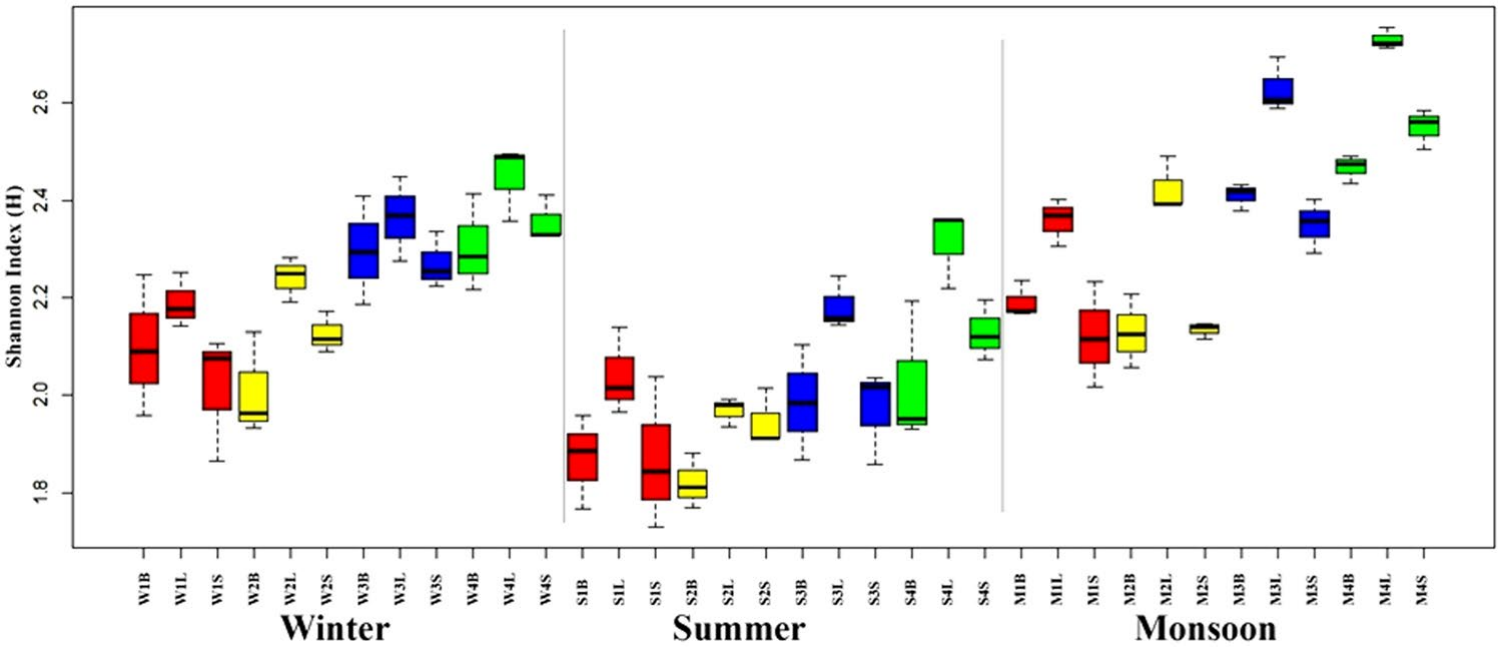
Boxplot representation of variability of species diversity (H) with respect to location, season and tissue type. The first letter of the three-letter acronyms denotes the season (W, winter; S, summer; M, monsoon), the second the location and the third the tissue type (B, barks; L, leaf; S, stem).

**Figure 3:**
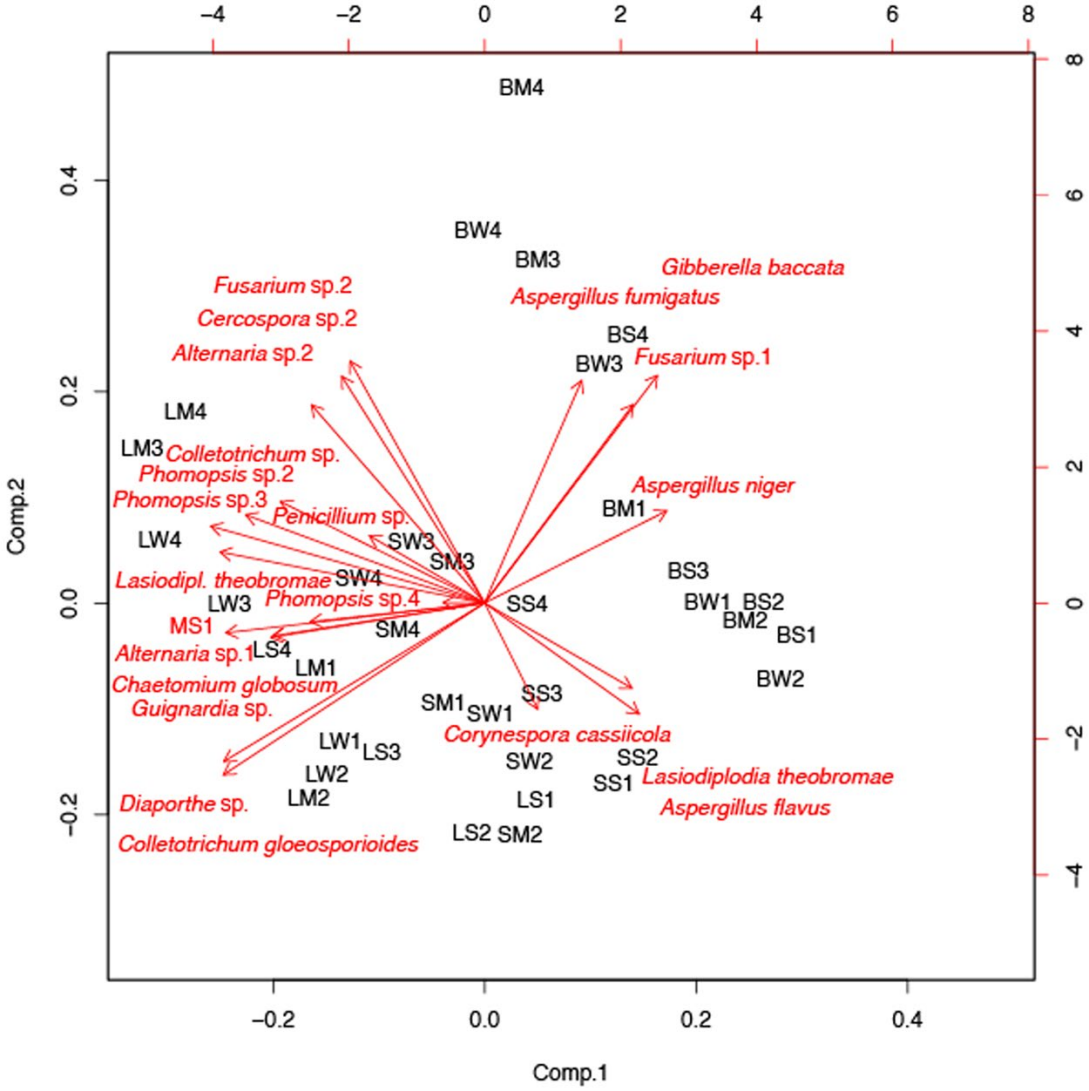
Biplot (Principal components analysis) depicting the relationship between fungal endophyte taxa (arrows) and sampling units (tissue type, season and location). The longer the arrow the more influence has the fungus connected with it, and the further removed a sampling unit gets positioned from the centre the stronger its influence. The closer a sampling unit and a fungal taxon the stronger is their relationship. The first letter of the three-letter acronyms denotes the tissue type (B, barks; L, leaf; S, stem), the second the season (W, winter; S, summer; M, monsoon), and the third the location.

Season, site and tissue type significantly influenced diversity of fungal endophytes (p ≤ 0.001) as summarised in Table 3. Shannon-Wiener index varied significantly (p ≤ 0.001) with season, location and tissue type (Fig. 2 and Table 3). Tissue type had the strongest effect (p ≤ 0.001) on species evenness of the endophytic fungal assemblage followed by location (p ≤ 0.05) (Fig. 3 and Table 3). CF (colonization frequency) increased from the driest location 1 (Loc1, Banaras Hindu Uunivesrsity campus, Uttar Pradesh) to the wettest location 4 (Loc4, Rajiv Gandhi University campus, Arunachal Pradesh) in all season. CF was significantly (p ≤ 0.001) lower in bark than in leaves (Fig. 2 and Table 4).

**Table 3:** Summary of MANOVA of different diversity indices.

Indices	Season (F2, 72)	Location (F3, 72)	Tissue (F2, 72)	Season × Location (F6, 72)	Season × Tissue (F4, 72)	Location × Tissue (F6, 72)	Season × Location × Tissue (F12, 72)
**Shannon** (**H′**)	180.857^***^	80.500^***^	59.126^***^	1.808 ^ns^	2.481 ^ns^	1.764 ^ns^	0.651 ^ns^
**Evenness**	1.463 ^ns^	3.715^*^	19.131^***^	1.282 ^ns^	0.817 ^ns^	2.047 ^ns^	1.524 ^ns^
**Species richness**	187.583^***^	104.233^***^	91.291^***^	4.087^***^	3.621^**^	1.738 ^ns^	1.350 ^ns^

***Significant at p ≤ 0. 001, **Significant at p ≤ 0.01, *Significant at p ≤ 0.05, ^ns^non-significant; where F is degree of freedom for different variables.

**Table 4:** Diversity parameters for aggregate data based on location, season and tissue.

Diversity Parameters	Loc1	Loc2	Loc3	Loc4	Winter	Summer	Monsoon	Bark	Leaf	Stem
Morphotypes (S)/Species richness	23	23	28	29	44	31	43	27	30	23
Individuals	1053	1176	1335	1525	1780	1298	2011	1413	2023	1653
Dominance (D)	0.1132	0.1055	0.09357	0.08807	0.09672	0.1198	0.08182	0.124	0.1073	0.1242
Shannon (H′)	2.51	2.56	2.74	2.81	2.79	2.54	2.95	2.59	2.63	2.44
Evenness (e^H/S)	0.5345	0.5619	0.5526	0.5723	0.369	0.4074	0.4441	0.4918	0.4613	0.5003
Colonization frequency (% CF)	75.3	65.9	58.07	52. 0	65.9	48.1	74.5	52.3	74.9	61.2

### A more detailed consideration of each factor is given below

#### Effects of geographical location

Highest number of endophytic isolates were recovered from samples of Loc4 (1525 isolates, 29 morphotypes) followed by location 3 (Loc3, Ranibagh, Uttarakhand) (1335 isolates, 28 morphotypes), Loc2 (1176 isolates, 23 morphotypes) and Loc1 (1053 isolates, 23 morphotypes) (Table 4). In term of colonization frequency Loc4 showed 75.3% followed by Loc3 with 65.9%, Loc2 with 58.07% and minimum at Loc1 with 52.0% (Table 4). Loc4 achieved the highest Shannon-Wiener (*H*′) index (2.81), followed by Loc3 (2.74), Loc2 (2.56) and Loc1 (2.51) (Fig. 2, Table 4). Different diversity parameters recorded in different locations, seasons and tissues is given in Table 4.

#### Effects of season

Seasonal effect was prominent with the maximum colonization occurring in monsoon (2011 isolates, 43 morphotypes) followed by winter (1780 isolates, 44 morphotypes) and the minimum in summer (1298 isolates, 31 morphotypes) (Table 4). CF (%) value varied with season with the highest in monsoon (74.5%) followed by winter (65.9%) and summer (48.1%) (Table 4). The relative abundance of most species was the highest in monsoon followed by winter and summer. Shannon-Wiener index was found as high as 2.95 in monsoon. Even the lowest index value recorded in summer (2.54) indicates good endophyte diversity in teak tree (Table 4).

#### Effects of tissue type

Of the different tissues sampled, leaf harboured maximum endophytes in terms of both total isolates (2023) and morphotypes (30). From stem tissue, 1653 isolates belonging to 23 different OTUs were recovered, whereas 1413 isolates and 27 morphotypes were obtained from bark. Leaf showed highest CF (74.9%) followed by stem (61.2%) and bark (52.3%). Leaf scored highest Shannon-Wiener index (2.63) followed by bark (2.59) and stem (2.44) (Table 4). Leaf also scored highest Shannon-Wiener index across all seasons and sites (Fig. 2).

## Discussion

### Diversity of endophytic mycobiota of *Tectona grandis*

The ITS1-5.8 S-ITS2 region of the rDNA is the most widely used marker for bar coding of fungi because it is considered to appropriately discriminate species^17^. However, in this study some of the morphotypes had identical sequences, although morphological differences were strong enough to keep them as separate species. Insufficiency of ITS sequences to separate fungal species has also been reported for other fungal species complexes^18, 19^. In contrast, intra-generic diversity of the ITS region of *Diaporthe* (*Phomopsis*) was high leading to the recognition of seven different species. High species diversity in this genus is in accordance with previous findings^20, 21^. Diversity of endophytic fungi in *Tectona* was found to be lower than in other tropical woody trees^22–24^. Inhibition of slow growing endophytes by the fast growing *Phomopsis* species might be one of the reasons for the low number of species detected in this study. The use of next generation sequencing (NGS) would probably lead to the discovery of many other species^25–27^. Predominance of ascomycotina in teak is in accordance with most studies done about endophytic assemblages in other plant species, so far^28, 29^, including tropical trees, e.g. *Terminalia arjuna* ^24^ and *Madhuca indica* ^30^. Interestingly, overall CF (62.83%) in teak tree was found low while many previous studies reported very high level of colonization frequency ranging from 95 to 100% in other trees^20, 25, 31^. The low CF and morphotypes might be due to the fact that the present study was carried out in different seasons, site and tissue types, unlike earlier studies limited to foliar tissue or favourable season which support highest CF. The reason for fewer morphotypes recovery may be also due to inability of fungi to overcome the physical and chemical barriers of the host plant^32^. Fungi easily colonise low-density wood compared to solid and high density wood, as found in teak. Teak wood also has high lignin content as another barrier for fungal colonization. Furthermore, flavonoids (rutin and quercitin) present in teak leaves play important roles in resisting fungal invasion^33^. The extract of hardwood sawdust of *T*. *grandis* has shown great inhibitory activity against several brown rot and white rot fungi. The naphthoquinone derivatives found in teak heartwood and leaves act as strong anti fungal agent^34^. Again, in a study teak leaf extract suppressed significantly the growth and spore formation of *Arthrinium phaeospermum* exhibiting its anti fungal property^35^. Furthermore, culture-dependent methods make it extremely difficult to isolate and enumerate biotrophic and unculturable species^36^ leading to lower CF and morphotypes estimate. This might be one of the reasons for the isolation of fewer basidiomycotina in the endophytes of *T*. *grandis*. Currently researchers prefer culture independent next generation sequencing (NGS) approaches to reveal the real endophytes diversity. However such methods are not error proof and have been blamed for overestimation^21, 36–38^. Serious doubts arise when predominating species or communities found in culture based isolation are completely absent^39^ or differ^26^ in NGS methods. Some studies which compared the traditional culture based endophyte diversity data with NGS data of the same host tree revealed that culture-based technique alone can reveal real qualitative picture of fungal endophytes^27, 36^. Furthermore, culture technique is the only way to get isolates for future wet lab use and to improve reference taxonomic database. In view of the above, it can be concluded that a combination of culture-dependent and culture-independent approaches need to be used for reliable estimate of endophyte diversity^37^. *Colletotrichum gleosporioides*, *Colletotrichum* sp. (*Colletotrichum siamense*), *Phomopsis* spp. and *Fusarium* sp.1 (*Albonectria rigidiuscula*) were ubiquitous and dominant at all four sites. Their dominance increased from Loc1 to Loc4. Perhaps to more favourable abiotic and biotic conditions for fungal growth, such as the prolonged duration of rain fall, greater annual precipitation, favourable temperature (Table 5), higher relative humidity, lesser anthropogenic perturbation and higher inoculum dose, tend towards optimum from Loc1 to Loc4. Above species along with *Fusarium* spp., *Alternaria* spp., *C*. *globosum*, *Aspergillus* spp., *Corynespora cassiicola*, *Lasiodiplodia* spp., *Hypoxylon fragiforme* and *Curvularia* sp. are well established endophytes of teak and other host plants^15, 16, 30, 39, 40^. Species diversity and dominance of *Diaporthe* (*Phomopsis*) species in *T*. *grandis* is similar to the findings of Mekkamol^15^ and Chareprasert *et al*.^16^. This finding added in establishing the *Diaporthe* (*Phomopsis*) spp. as consistent teak endophytes which might have co-evolved with teak^41^. However, *Diaporthe* (*Phomopsis*) spp. have also been reported as the dominant endophyte in other trees like *Azadirachta indica* ^42^, *Luehea divaricata* ^43^ and *Madhuca indica* ^30^. Dominance of *Diaporthe* (*Phomopsis*) spp. in *Trichilia elegans* in a recent report also supported our finding^20^. Of 45 morphotypes, 17 showed dominance <0.5%. Presence of such a large number of species in low colonization frequency clearly suggests the adequacy of sample size as well as isolation protocol. The presence of rarely reported fungal endophytes, such as, *Coprinellus*, *Cercospora*, *Ceratobasidium*, *Periconia* and *Pyrenochaeta* also suggests that sampling and isolation methods were satisfactorily employed. These endophytes seem to be reported for the first time from the Indian subcontinent. Previous studies have shown that colonization of endophytes is strongly affected by geographic location, climatic conditions, seasonal changes, host and host tissues^44, 45^. Keeping in view the suggestions of Arnold *et al*.^3^ that host preference and spatial orientation of tropical tree mycobiota can be better explained by their relative colonization frequency rather than presence or absence of morphospecies, we analysed our data for the same. Significant statistical differences in diversity measures were observed along locations, seasons and tissue types in MANOVA (p ≤ 0.001), based on Shannon-Wiener index (*H′*) and species richness data (Table 3). Biplot analysis done to analyse interactive effect of all the three variables on dominant endophytes (%D ≥ 0.5) showed tissue type based grouping of isolates indicating its strongest effect (Fig. 3).

**Table 5:** Geographic coordinates, climatic conditions and unique characteristics of study sites.

Site	Geographic coordinates	*Seasonal rainfall during the study period in mm (Jan 2011–Dec 2011)			*Monsoon period in study year		*Seasonal mean of minimum and maximum T (in °C)		Uniqueness and reasons for selection as study site
		Winter	Summer	Monsoon	Onset	Withdrawal	Mean Min.	Mean Max.	
Loc1: (BHU) Banaras Hindu University, Uttar Pradesh	25°16′34.93″N 82°59′20.22″E	8.5	247.3	844.5	17 June	30 Sept.	8.8 (W), 16.3 (S), 20.7 (M)	29.3 (W), 40.9 (S), 33.8 (M)	Located in the highly fertile Gangetic plain of India
Loc2: (HN) Hathinala, Uttar Pradesh	24°18′0.97″N 83°6′29.96″E	9.5	267.8	1094	17 June	30 Sept.	11.1 (W), 22.69 (S), 22.65 (M)	25.82 (W), 37.44 (S), 31.24 (M)	Located in the dry deciduous tropical forest of Vindhayan region
Loc3: (RB) Ranibagh, Uttrakhand	29°17′31.97″N 79°32′34.23″E	97.8	462.90	1460.5	20 June	26 Sept.	1.7 (W), 7.5 (S), 9.7 (M)	15.4 (W), 23.5 (S), 21.6 (M)	Located in the biodiversity rich Himalayan foot hills with prevailing low temperature
Loc4: (RGU) Rajiv Gandhi University, Arunachal Pradesh	27°8′52.34″N 93°45′53.44″E	51.5	1158.1	1948.3	8 June	13 Oct.	15 (W), 20.4 (S), 22 (M)	21(W), 34.2 (S), 30.2 (M)	Located in one of the world’s top mega diversity centre, world’s northernmost tropical rain forest, receives almost heaviest annual precipitation worldwide

(*Source: National Data Centre, IMD, Pune, India). W = Winter, S = Summer, M = Monsoon.

### Effects of spatial variation

Earlier studies have supported the view that climate and rainfall pattern of the investigation area strongly affect the fungal assemblage^45^. Loc4 in Arunachal Pradesh registered highest annual precipitation followed in decreasing order by the Loc3 in Uttrakhand, Loc2 at Mirzapur in Uttar Pradesh and Loc1 at Varanasi in Uttar Pradesh during the year of study. The decreasing patterns of annual rainfall from Loc4 to Loc1 exactly matched the decreasing trend for diversity and species richness at these sites. Diversity index and species richness were maximum at Loc4, is in concordance with the favourable conditions found there for fungal growth and dispersion as region is attributed with tropical climate accompanied with heavy and comparatively long spanned annual rain fall^46^. Greater species richness of pine trees in autumn compared to spring due to higher rainfall in the former also highlights the role of rainfall in shaping fungal assemblage^47^. Vaz *et al*.^48^ found significant statistical difference in colonization of fungal endophytes with increase in precipitation and decrease in temperature. Higher diversity at Loc3 in comparison to Loc1 and Loc2 might be due to higher relative humidity, as reported in the case of *Trichilia elegans* ^20^. No doubt, factors other than rainfall and humidity also affect the diversity of endophytic assemblage. We also observed location specific distribution of certain endophytes at least at species level. *Aspergillus* sp.1 (*Aspergillus viridinutans*) was specific to Loc1, likewise *Coprinellus* sp., *Ceratobasidium* sp., and *Fusarium* sp.2 (*Fusarium incarnatum*) were exclusively isolated from Loc3 whereas *Cercospora* sp.1 (*Cercospora gerberae*), and *Curvularia* sp. were restricted to Loc4. Space limited distribution of these taxa indicates spatial structuring of endophytic communities.

### Effects of temporal variation

Rain splashes help in dissemination of inoculum materials, and high humidity and low temperature support fungal spore germination and multiplication. These conditions lead to high infection rate and fungal establishment in monsoon and winter seasons. Contrary to this, ambient environment exhausted of potential inocula in summer resulted in little horizontal transfer of endophytes^38^. Further, under stress conditions host plants develop different structural modifications and defense chemicals leaving lesser access to intruders. We found colonization frequency of most endophytic species maximum in monsoon followed by winter and summer. Further, increase in colonization of *A*. *flavus*, *Aspergillus niger* and *Guignardia* sp., which are considered as xerophilic fungi, in summer indicates seasonal effect on mycobiota assemblage. These fungi face lesser competition from other humidity requiring fungi resulting in greater colonization during dry period. On the other hand, *Phomopsis* producing a large number of slimy spores in rainy season may reduce the chances of xerophilic fungal growth during monsoon period. The present seasonal findings are in accord with the earlier observations^16, 40^. The highest CF in monsoon and the greatest species richness in the winter is agree with the findings of Chareprasert *et al*.^16^ in *T*. *grandis*. Guo *et al*.^49^ also reported highest endophyte CF in spring in needle of *Pinus tabulaeformis*. On the other hand, Collado *et al*.^50^ reported highest species richness in spring and Helander *et al*.^51^ reported no seasonal effect on colonization frequency of endophytes in old needles of Scots pine but in young needle CF increased in summer.

### Effects of tissue type

Maximum colonization frequency and species richness in leaves might be due to their large size (20–50 cm × 15–40 cm) which offers considerably great inoculum capture area, abundance of natural openings in the form of stomata, hydathodes and glandular openings as easy entry points, and hairy surface helpful in inoculum landing and attachment. Tenderness of leaf, in contrast to bark and stem tissues, helps endophytes in easy access to internal tissue. The present findings of maximum colonization frequency and species richness in leaves corroborates the earlier findings^15, 16^. The highest diversity in *Tectona* leaf is supported by the presence of as many as 22 species of endophytic fungi in a single leaf of tropical tree *Manilkara bidentata* ^52^. Indian medicinal herb *Tinospora cordifolia* ^40^ tissues also harbours endophytes like *Tectona* (leaf > stem > bark) while *A*. *Indica* ^42^ showed slightly different results where endophytes recovered maximally in leaf followed by bark and stem. Gond *et al*.^53^ reported maximum endophyte colonization in bark of *Aegel marmelos* while Verma *et al*.^30^ reported greatest recovery of endophytes from stem tissue of *M*. *indica*. The above findings lead to the conclusion that different tissues of various plants support endophytes colonization at varying rate. Reports on strict host specificity and preference are very rare^44, 54^ however, isolation of *C*. *globosum* strictly from leaf tissue across all four sites in all three seasons, indicated its remarkable specificity towards leaf in the present study. This finding is also supported by host specificity of *Chaetomium* for *T*. *arjuna* ^24^. Besides *C*. *globosum*, *Guignardia* sp. (*Phyllosticta elongata*) and *Paecilomyces* sp.1 (*Paecilomyces variotii*) also showed strong affinity towards leaf tissue as they were isolated uniformly but exclusively from leaf sample of at least three distantly located sites in different seasons. Sun *et al*.^55^ reported significant host and tissue preference in *Betula platyphylla*, *Quercus liaotungensis* and *Ulmus macrocarpa*. Ek-Ramos *et al*.^56^ also reported tissue specificity of some endophytes in *Gossypium hirsutum*. Fungal endophytes associated with three South American Myrtaceae members also exhibited preferences in the colonization at leaf level^48^. In this study stem comes second in terms of CF but with less species richness and diversity than bark. Minimum endophytes CF in bark might be due to its dead rhytidome but direct exposure to the external environment supports it to harbour greater species richness than stem. Since different plant tissues have distinct anatomy and functions, these may have influenced the dominance of dissimilar fungal groups in different tissue types in the present study. Effect of tissue type on endophyte colonisation has also been highlighted in many earlier studies^40, 55–57^. Host-dependent structuring of endophytic components can also be explained by the fact that being inside the host they never experience external environmental fluctuations directly. But we can not wholly discount the role of external environment, e.g. during infection, as it would influence the fungal inocula type and density.

Since bark and stem are permanent organs of the plant and do not show significant changes in their shape and size within a year, the age of these organs during the study does not seem to have exerted significant influence. However, the leaves are deciduous and their age, toughness, shape and chemistry tremendously vary within a year. New leaves of *T*. *grandis* appear in March (spring), grow and mature up to September (monsoon) and senesce thereafter until partial fall in January (winter). For the leaf, minimum diversity was recorded in summer and maximum in monsoon. But it cannot be ascertained that this trend was solely due to leaf age and its chemistry as Arnold and Herre^58^ found no observable effect of leaf age on endophyte colonization in *Theobroma cacao* with all young and mature leaves equally colonisable. However, leaf age may be considered as an additional factor along with seasonal and geographic factors for variations in diversity.

In conclusion, all the three variables, namely, tissue type, season and location, effected the fungal endophyte composition of *T*. *grandis* with tissue type having the predominant effect. *T*. *grandis* has supported moderate endophytic fungal diversity. This diversity estimate can further be augmented by using both culture-dependent and culture-independent approaches.

## Methods

### Sites and sample acquisition

Four different sampling sites in northern and north-eastern India differing in climatic and geographic conditions were selected for the study (Table 5). Loc1 is situated in the sub-tropical eastern Gangetic plains, Loc2 is situated in the dry deciduous tropical forest of the Vindhayan region, Loc3 is situated in the foot hills of Himalaya with mild summer and low winter (mean minimum temperature 1.7 °C) temperature and Loc4 is atop Rono hills situated in one of the global biodiversity hot spot region with heavy annual precipitation.

Mature, green, asymptomatic leaves in triplicate were randomly collected from the lowermost branches of five *T*. *grandis* trees. Bark and stem samples were taken at chest height (1.37 m, to maintain sampling consistency). Sampling was done at every study sites in three season namely winter (January), summer (May) and monsoon (September) in 2011. At every occasion sampling was done from the same trees. Collected plant parts were packed separately in polybags and kept in icebox (4 °C) for transportation and further processing.

### Surface sterilization and endophyte recovery

The surface sterilization method proposed by Petrini *et al*.^59^ was adopted to *T*. *grandis* tissues and verified by ‘leaf imprint method’ of Schulz *et al*.^60^ Briefly, 5 cm × 5 cm piece from each leaf, and 5 cm × 2 cm section from each bark and stem sample were cut out and cleansed in running tap water followed by sequential dipping in 70% ethanol (2 min), 4% sodium hypochlorite (4 min for bark and stem, 2 min for leaf), 70% ethanol (2 min) and finally rinsed thrice with sterile deionised water to remove the surface sterilents. After air drying in laminar flow, the samples were cut into small pieces (5 mm × 5 mm) under strict aseptic conditions. About 4–5 segments were placed on each Petri dish containing potato dextrose agar medium (PDA, HiMedia) supplemented with 150 μgml^−1^ of streptomycin sulphate (HiMedia). Selection of PDA, which supported maximum endophytes recovery in terms of both number and types, was done over two other media namely Czapek Dox agar (CDA) and malt extract agar (MEA) after screening 50 segments of each tissue types as details given in Table 6. A total of 225 segments of each tissue type in each season from each location were screened for endophyte presence. Thus, seasonally 2700 segments were examined in this study, amounting to a total of 8100 segments. The Petri dishes were sealed with Parafilm (Bemis Flexible Packaging, USA) and incubated at 27 ± 2 °C in BOD cum humidity incubator (Calton, NSW, New Delhi, India) under 12 h light and dark cycle and monitored every day for fungal emergence up to one month. Different endophytes emerging from the explants were transferred and maintained on fresh PDA plates as axenic cultures.

**Table 6:** Preliminary fungal endophytes isolation for suitable culture medium selection.

Endophytes	Czapek Dox agar			Malt extract agar			Potato dextrose agar		
	Bark	Leaf	Stem	Bark	Leaf	Stem	Bark	Leaf	Stem
*Alternaria* sp.	0	0	0	1	0	1	3	2	2
*Chaetomium globosum*	0	0	0	0	1	0	0	2	0
*Colletotrichum gloeosporioides*	0	1	0	0	1	0	0	3	0
*Fusarium baccata*	0	0	0	0	0	0	2	3	0
*Fusarium* sp.	0	0	1	1	0	3	4	0	6
*Pseudofusicoccum adansoniae*	0	1	0	0	3	0	2	4	2
*Diaporthe* sp.	2	2	1	3	4	3	4	8	6
*Diaporthe longicola*	3	5	4	4	7	6	6	9	9
*Phomopsis* sp.	0	0	0	2	0	0	6	0	0
Total isolates	5	9	6	11	16	13	27	31	25
Grand total isolates	20			40			83		
Total morphotypes	5			8			9		

### Macroscopic and microscopic identification of recovered fungal endophytes

Based on the culture characteristic, such as, shape, size, colour, texture, growth pattern and back side colour of colony and microscopic details, all the fungal isolates were grouped into 45 distinct morphotypes/OTUs. Sporulating cultures were examined using a camera-coupled Nikon Trinocular Light Microscope (Model E-600). Standard taxonomic manuals were used for the morphological identification and grouping of fungal isolates^61, 62^. After identification, representatives of each morphotype were preserved and stored in triplicates at the Department of Botany, Banaras Hindu University, Varanasi, India.

### Molecular identification of endophytes

#### Total genomic DNA extraction

The total genomic DNA of all morphotypes, except four well known, was extracted following the lab developed amended SDS-CTAB protocol to get high throughput fungal DNA extraction. In short, 0.3 g pure mycelium was pulverized with plastic micro-pestle in 500 µl lysis buffer in a microcentrifuge tube. If needed, more lysis buffer was added to maintain viscosity of the paste. This step was followed by the addition of 75 µl of 10% SDS solution. After gentle mixing, the tube was placed in shaking water bath at 37 °C for 1.5 h with intermittent gentle inversion followed by addition of 80 µl of each 5 M NaCl and CTAB/NaCl solutions and incubation at 65 °C in water bath for 45 min at 60 rpm. DNA was extracted by addition of equal volume of phenol:chloroform:isoamyl alcohol (25:24:1) and centrifugation at 12000 rpm for 15 min. Aqueous supernatant was transferred to fresh centrifuge tube and DNA was precipitated by addition of equal volume of ice chilled isopropanol at −20 °C. After 12 hr of incubation, the tube was centrifuged at 12000 rpm for 15 min. The pellet obtained was washed with 70% ethanol and air dried in laminar flow before re-suspending in 20–30 µl of TE buffer. Isolated DNA was electrophoresed on 0.8% (w/v) agarose gel stained with ethidium bromide (0.5 μgml^−1^) and visualized under UV transilluminator.

#### PCR amplification and DNA sequencing

The universal primer pair ITS1 (5′ TCCGTAGGTGAACCTGCGG 3′) and ITS4 (5′ TCCTCCGCTTATTGATATGC 3′) were used to amplify the fungal nrITS regions of representative isolate of each morphotype^63^. PCR amplifications^64^ (modified) were performed on programmable S1000 Thermal Cycler (BIO-RAD) in 50 µl reaction volumes, each having 0.4 µl of 5 Uµl^−1^ *Taq* DNA polymerase (Genei, India), 1 µl of 10 mM dNTPs (Genei, India), 2 µl of each primer (10 pM, Eurofins genomics, India), 5 µl of 10X PCR buffer with MgCl_2_, 37.6 µl ultrapure water and 2 µl extracted DNA template. Optimized condition used for amplification was pre-denaturation at 95 °C for 5 min followed by 35 cycles of each denaturation at 95 °C for 1 min, primer annealing at 54 °C for 1 min, extension at 72 °C for 1 min and at last 1 cycle of final extension at 72 °C for 5 min. A non-template negative control was also run each time. Integrity and quality of resulting PCR products were examined on 1.5% (w/v) agarose gel impregnated with ethidium bromide (0.5 μgml^−1^) and visualized under a UV transilluminator. Amplified ITS fragments were cleaned by HiYield Gel/PCR DNA mini kit (Real Biotech Corporation) strictly following manufacturer manual and sequenced (Applied Biosystems 3130 Genetic Analyser) at Interdisciplinary School of Life Sciences (ISLS), BHU, India using ITS1 (forward) primer and BigDye Terminator v3.1 Cycle Sequencing Kit.

#### Blast search and phylogeny analysis

For phylogenetic analysis sequences were nBLAST searched against the NCBI database (blast.ncbi.nlm.nih.gov) and trimmed to cover the entire region of closest reference sequence. These trimmed sequences were further used to get the closest taxonomic match and phylogenetic tree construction. Multiple sequence alignments of 41 OTUs and 2 closest named reference sequences for each morphotype were done online by Clustal Omega (www.ebi.ac.uk/Tools/msa/clustalo/). The neighbor-joining phylogenetic and molecular evolutionary analyses were conducted using MEGA5. The evolutionary distances were computed using the Maximum Composite Likelihood method and are in the units of the number of base substitutions per site^65^. The obtained ITS sequences (unedited) were submitted to GenBank for accession numbers (www.ncbi.nlm.nih.gov/genbank). A detailed account of the sequenced OTUs with their respective GenBank accession number and the closest match (based on maximum % identity) are listed in Table 1.

#### Fungal diversity analysis

The percent colonization frequency (%CF) was calculated^66^ as %CF = (N_col_/N_t_) × 100, where, N_col_ = number of plant tissue segments colonized and N_t_ = total number of plant tissue segments examined. Percentage dominance (%D) of each OTU was calculated as %D = n/N × 100 where, n = total isolates of an OTU, N = total isolates of all OTUs. Shannon-Wiener index (*H′*) was calculated for each season, location and tissue type using PAST software (http://folk.uio.no/ohammer/past/). Variation in Shannon-Wiener index (*H′*) with season, site and tissue is depicted in the form of boxplots drawn with the help of “R” statistical software (R Development Core Team 2009)^67^. Effects of season, site and tissue type on distribution and diversity of fungal endophytes were analysed by multivariate linear model analysis (MANOVA) and bi-plot analysis using SPSS v16 and R v2.15.1 (R Development Core Team 2009)^67^ software, respectively. For MANOVA, season, site and tissue type were considered as independent factors while Shannon–Wiener index, species richness and evenness were treated as dependent factors or response variables. Principal components analysis was done to reveal the interaction between fungal endophytes with sampling variables (tissue type, season and location) using R statistical software.
